# Impact of Donor Age on Graft Failure After Deceased Donor Liver Transplantation by Donor-Recipient Sex Combinations: An Analysis of the UNOS OPTN Database

**DOI:** 10.3390/jpm15080357

**Published:** 2025-08-05

**Authors:** Sangbin Han, Vatche A. Agopian, Justin A. Steggerda, Irene K. Kim, Alison Sanford, Yi-Te Lee, Ji-Hye Kwon, Jin Soo Rhu, Gaab Soo Kim, Ju-Dong Yang

**Affiliations:** 1 Department of Anesthesiology and Pain Medicine, Samsung Medical Center, Sungkyunkwan University School of Medicine, Seoul 06351, Republic of Korea; 2 Department of Surgery, David Geffen School of Medicine UCLA, Los Angeles, CA 90095, USA; 3 Department of Surgery, Cedars-Sinai Medical Center, Los Angeles, CA 90048, USA; 4 Division of Gastroenterology and Hepatology, Cedars-Sinai Medical Center, Los Angeles, CA 90048, USA; 5 Department of Surgery, Samsung Medical Center, Sungkyunkwan University School of Medicine, Seoul 06351, Republic of Korea

**Keywords:** estrogen, infertility, steatosis, perimenopause, sex dimorphism, sex disparity, sex hormone, estrogen receptor

## Abstract

**Background** Sex disparity has been highlighted in personalized medicine for various human diseases including acute/chronic liver diseases. In the transplant community, greater graft failure risk in female-to-male liver transplantation (LT) has been repeatedly reported, and a recent study in living donor LT reported that the inferiority of female-to-male LT is shown only when donor age is ≤40 y. We aimed to analyze the United Network for Organ Sharing (UNOS) database to test if the poorer outcome of female-to-male LT changes by donor age of 40 y in deceased donor LT, as shown in living donor LT. **Methods** In this retrospective cohort study, 11,752 adult patients in the UNOS registry who underwent deceased donor LT between 2000–2023 were analyzed. Multivariable analysis was performed to adjust the effects from transplant years, graft ischemia time, disease severity, and others. The primary outcome was graft failure. **Results** Within the subgroup of recipients with ≤40 y donors, graft failure risk was significantly greater in female-to-male LT than others (vs. female-to-female, HR = 1.43 [1.16–1.76], *p* < 0.001; vs. male-to-female, HR = 1.46 [1.18–1.81], *p* < 0.001; vs. male-to-male, HR = 1.26 [1.16–1.49], *p* = 0.009). In contrast, within the subgroup of recipients with >40 y donors, the risk was comparable between female-to-male LT and other donor-recipient sex groups (vs. female-to-female, *p* = 0.907; vs. male-to-female, *p* = 0.781; vs. male-to-male, *p* = 0.937). We tested various cutoff donor ages and determined that 40 y is the best cutoff value to define the risk subgroup in female-to-male LT. **Conclusions** In the current study, we found that the sex disparity shown in living donor LT is also observed in deceased donor LT. That is, post-transplant graft failure risk was greater in female-to-male LT than other donor–recipient sex groups only when donor age was ≤40 y. In contrast, graft failure risk was comparable irrespective of donor-recipient sex combinations when donor age was >40 y.

## 1. Introduction

Sex disparity has been seen in various human diseases, like cardiovascular, oncologic, metabolic, immunological, and neurological diseases [1,2,3]. Although the mechanisms underlying the sex disparity are thought to be related to genetic/hormonal factors as well as lifestyle, large parts are still unknown [4]. As personalized medicine advances, the importance of examining sex disparity and underlying mechanisms is now being highlighted not only for the prevalence or diagnosis but also for targeted-treatment and post-treatment prognosis [1], with an increased focus on sex-based considerations to promote personalized, sex-specific healthcare [2,4]. This phenomenon is also observed in the liver, including chronic disease like alcoholic liver disease and hepatocellular carcinoma (HCC) as well as acute ischemia-reperfusion injury [5,6,7,8], while hepatic estrogen signaling is thought to be deeply involved [6,8,9]. In addition, the risk of post-transplant liver graft failure is known to be greater in male recipients receiving a graft from a female donor, so-called female-to-male liver transplantation (LT) [10]. Since the landmark study by Kahn et al. [11], clinical studies have consistently demonstrated inferior outcomes of female-to-male LT [12,13,14,15], while recent research validated it in living donor LT [9,16]. Our research team further found that the poorer outcomes of female-to-male LT did not result from surgical issues like size mismatch in the vessels, bile duct, and graft (e.g., graft-to-recipient weight ratio) between female donors and male recipients [10], supporting the presence of medical factors like post-transplant hepatic defeminization (or acute decrease in hepatic estrogen receptor) as suggested by previous studies [6,9,17]. In another recent study in living donor LT, we found a specific subgroup that the inferiority of female-to-male LT is shown. That is, the inferiority was abrogated when donor age was >40 y [18], further supporting that the anatomical mismatch is not the main factor of the poorer outcome of female-to-male LT. In this study, we aimed to evaluate whether the abrogation of greater risk of female-to-male LT with >40 y donor is also observed in a deceased donor population using the database of the United Network for Organ Sharing (UNOS) and Organ Procurement and Transplantation Network (OPTN).

## 2. Materials and Methods

**Subjects and data source**

**Figure 1:**
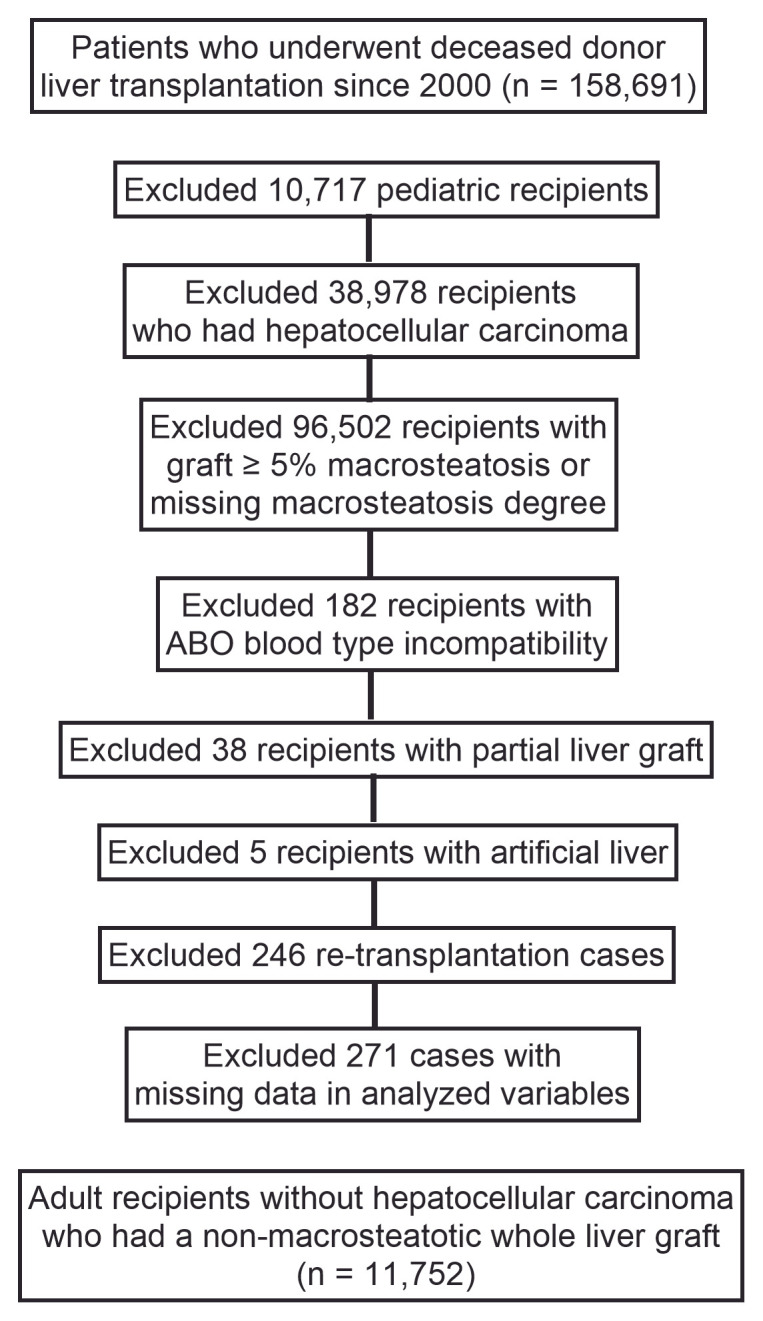
Subject selection flow diagram.

Among 3,567,269 patients registered in the waiting list from 1987 to 2023 in the UNOS data, we enrolled 158,691 patients who underwent deceased donor LT since 2000 in the study. The following subject selection process is shown in Figure 1. We excluded 10,717 pediatric recipients under 16 y. Among the remaining 147,974 recipients, 38,978 recipients who had HCC at transplantation were further excluded because hepatic estrogen signaling is known to impact differently on recipients with HCC and those without HCC [7,18,19]. Among the remaining 108,996 recipients, we only included 12,494 who received non-macrosteatotic graft (macrosteatosis degree < 5%) [20,21] because macrosteatosis changes the content of hepatic estrogen receptor and consequent hepatic estrogen signaling [6,22,23]. Because a large portion of subjects were lost due to missing macrosteatosis data, we added an analysis for the entire 108,996 recipients (Supplementary Figure S1). Patients who underwent ABO incompatible transplant (*n* = 182), who received a partial liver graft instead of the whole liver (*n* = 38), who were transplanted with artificial liver (*n* = 5), and who underwent re-transplantation (*n* = 246) were sequentially excluded. Among the remaining 12,023 recipients, we further excluded 129 patients with missing graft survival data, 41 patients with missing pretransplant dialysis data, and 101 patients with missing diabetes history. Finally, the remaining 11,752 adult non-HCC recipients who had a non-macrosteatotic whole liver graft from a decreased donor without previous LT history were included in the study.

All data analyzed in the study were derived from the OPTN data files created based on data as of 6 October 2023. The data have been supplied by the UNOS as the contractor for the OPTN. The interpretation and reporting of these data are the responsibility of the authors and in no way should be seen as an official policy of or interpretation by the OPTN or the U.S. Government. The database used and/or analyzed during the current study is available from the corresponding author on reasonable request. The Institutional Review Board of Samsung Medical Center approved this retrospective cohort study (SMC 2024-09-084) and waived the requirement for written informed consent. The study was conducted in accordance with the principles of the Declaration of Helsinki and Good Clinical Practice Guidelines.

**Statistics**

The primary outcome was post-transplant graft failure (death or re-transplantation). Survival analysis was performed using the Cox model while all patients were followed for a maximum of 10 y, and the results were described using hazards ratios (HR) with 95% confidence interval. The cutoff of donor age was set at 40 y based on previous studies [6,10,18]. To validate the cutoff age, a sensitivity test was performed to test various cutoff donor ages. We classified primary liver disease into the following 4 categories: viral, alcoholic, metabolic dysfunction-associated steatohepatitis (MASH), and others (Supplementary Table S1). As a more recent terminology, MASH was used in this manuscript instead of non-alcoholic steatohepatitis (NASH), the form described in the UNOS data [24]. All covariables analyzed in univariable analysis were entered into multivariable analysis. The continuous variables were described as median with interquartile range and categorical variables were expressed as frequency (%). All reported *p*-values were two-sided, and *p* < 0.05 was considered significant. Data were analyzed using SPSS 29.0 (SPSS Inc, Chicago, IL, USA).

## 3. Results

The primary liver disease of the 11,752 recipients were as follows: viral (*n* = 2211, hepatitis C = 1990, hepatitis B = 212, and hepatitis A = 9), alcoholic (*n* = 3840), MASH (*n* = 1909), biliary (*n* = 1166), metabolic (*n* = 374), autoimmune (*n* = 361), drug (*n* = 84), tumor other than HCC (*n* = 270), cryptogenic (*n* = 718), and miscellaneous (*n* = 819). For analysis, we grouped them into four categories: viral (*n* = 2211), alcoholic (*n* = 3840), MASH (*n* = 1909), and others (*n* = 3792). There were 2904 (24.7%) male recipients of grafts from female donors. Other recipients consisted of 2456 (20.9%) female recipients with female donors, 2099 (17.9%) female recipients with male donors, and 4293 (36.5%) male recipients with male donors. Clinical data of four donor–recipient sex groups of recipients with ≤40 y donor are described in Table 1 and the data of recipients with >40 y donor are described in Table 2.

**Analysis for recipients with ≤40 y donors**

**Figure 2:**
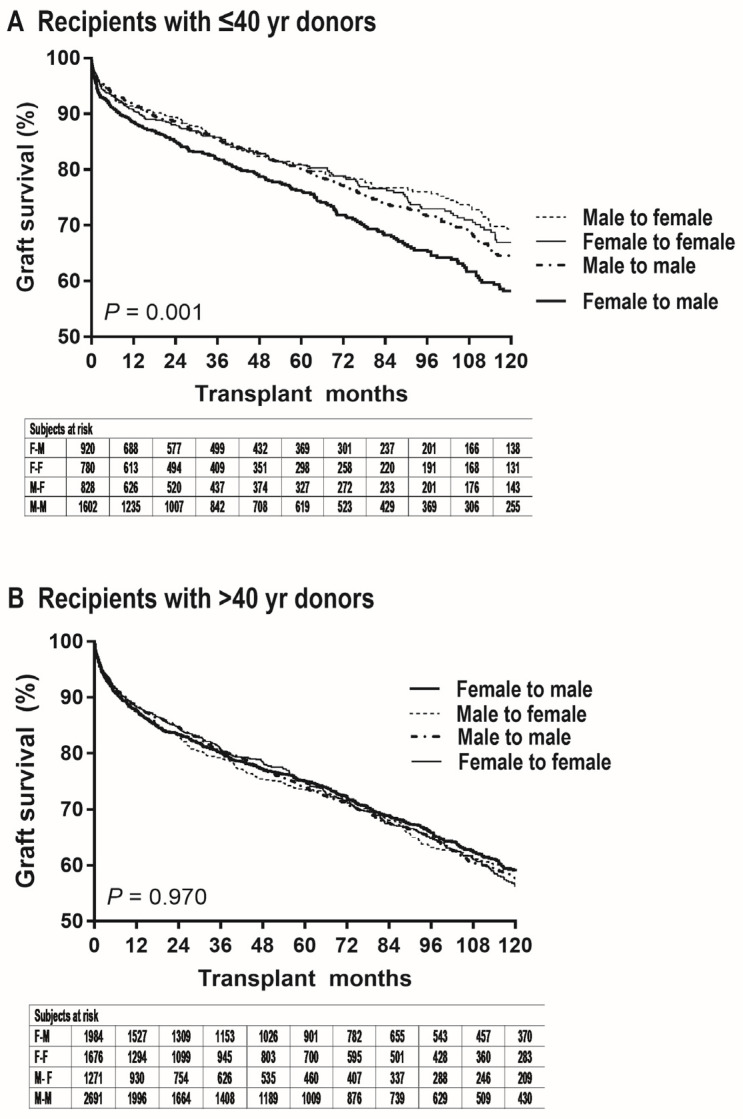
Graft survival according to donor/recipient sex in subgroup of recipients with (**A**) donors aged ≤ 40 y or (**B**) donors aged > 40 y.

The median follow-up time was 50 months with the interquartile range of 22 to 102 months. As shown in Figure 2A, graft failure risk was significantly greater in female-to-male LT than in other donor-recipient sex groups within the subgroup of recipients with ≤40 y donors (vs. female-to-female, unadjusted HR = 1.33 [1.10–1.62], *p* = 0.006; vs. male-to-female, HR = 1.41 [1.15–1.72], *p* < 0.001; vs. male-to-male, HR = 1.27 [1.08–1.50], *p* = 0.005). In female-to-male LT, the probability of graft survival after 1, 2, 5, and 10 y post-transplantation was 89.1% (86.8–91.0%), 85.4% (82.8–87.6%), 76.7% (73.4–79.6%), and 58.5% (53.6–63.1%), respectively. In female-to-female LT, the probability of graft survival was 91.0% (88.7–91.9%), 88.4% (85.8–90.6%), 81.2% (77.7–84.2%), and 67.2% (62.0–71.8). In male-to-female LT, the probability of graft survival was 92.2% (91.1–93.9%), 90.1% (87.7–92.1%), 81.7% (78.3–84.6%), and 69.9% (65.0–74.2%). In male-to-male LT, the probability of graft survival was 92.6% (94.1–93.8%), 89.3% (87.5–90.8%), 80.8% (78.3–83.0%), and 65.1% (61.4–68.6%), respectively. The proportion of graft failure at 1 y tended to be greater in female-to-male LT (vs. female-to-female, *p* = 0.165; vs. male-to-female, *p* = 0.049; vs. male-to-male, *p* = 0.007). The proportion of graft failure at 3 y was significantly greater in female-to-male LT (vs. female-to-female, *p* = 0.044; vs. male-to-female, *p* = 0.023; vs. male-to-male, *p* = 0.012). As shown in Table 3, the results of multivariable analysis demonstrated that female-to-male donation is an independent risk factor for graft failure within the subgroup of recipients with ≤40 y donors (vs. female-to-female, adjusted HR = 1.37 [1.12–1.69], *p* = 0.003; vs. male-to-female, HR = 1.43 [1.15–1.75], *p* = 0.001; vs. male-to-male, HR = 1.25 [1.06–1.49], *p* = 0.009). Consistent effect sizes showed the insignificant confounding effects from covariables and independence of the impact of female-to-male donation.

**Analysis for recipients with >40 y donors**

As shown in Figure 2B, graft failure risk was comparable between female-to-male LT and other donor-recipient sex groups within the subgroup of recipients with >40 y donors (vs. female-to-female, HR = 0.98 [0.87–1.11], *p* = 0.755; vs. male-to-female, HR = 0.98 [0.86–1.12], *p* = 0.780; vs. male-to-male, HR = 0.99 [0.89–1.10], *p* = 0.824). In female-to-male LT, the probability of graft survival after 1, 2, 5, and 10 y post-transplantation was 87.7% (86.1–89.1%), 83.4% (81.6–85.0%), 75.0% (73.8–77.0%), and 59.0% (56.0–61.8%), respectively. In female-to-female LT, the probability of graft survival was 88.4% (86.7–89.9%), 84.5% (82.6–86.2%), 74.9% (72.4–77.2%), and 56.2% (52.8–59.5). In male-to-female LT, the probability of graft survival was 87.2% (85.2–89.0%), 83.0% (81.7–85.1%), 73.5% (70.5–76.2%), and 59.5% (55.6–63.2%). In male-to-male LT, the probability of graft survival was 88.5% (87.2–89.7%), 84.7% (83.2–86.1%), 74.0% (72.0–75.9%), and 57.8% (55.1–60.4%), respectively. As shown in Table 4, the results of multivariable analysis demonstrated that female-to-male donation is not a risk factor for graft failure within the subgroup of recipients with >40 y donors (vs. female-to-female, HR = 1.02 [0.90–1.15], *p* = 0.762; vs. male-to-female, HR = 1.00 [0.88–1.15], *p* = 0.966; vs. male-to-male, HR = 0.99 [0.88–1.10], *p* = 0.827).

**Sensitivity test to validate the donor age of 40 y**

**Figure 3:**
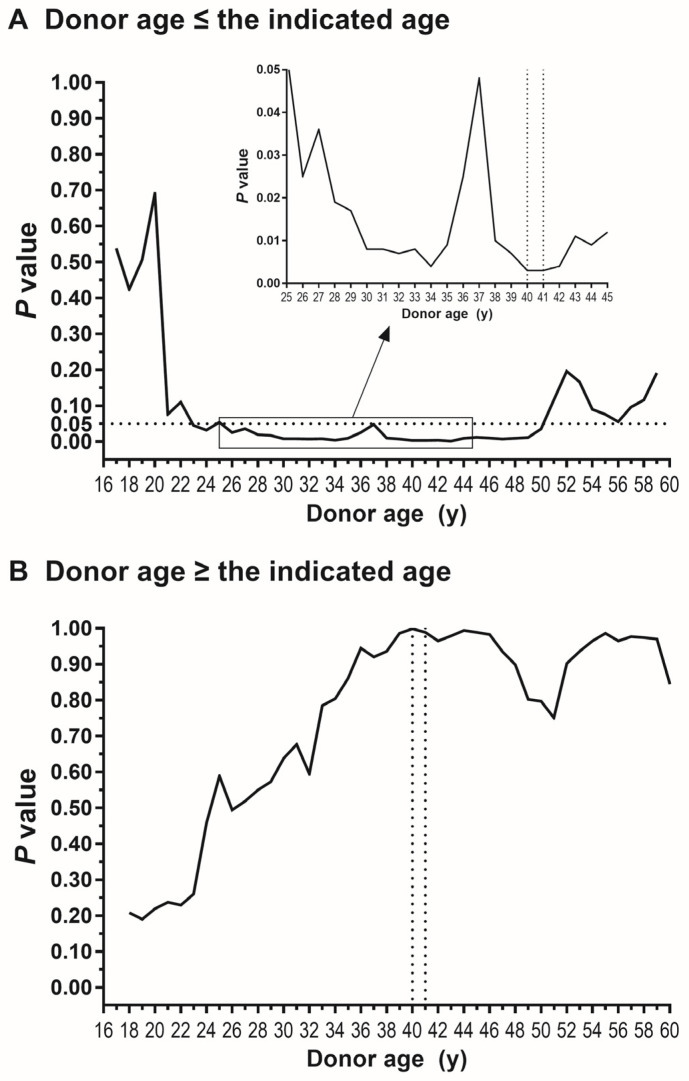
Statistical significance of inferiority of female-to-male donation regarding post-transplant graft failure (**A**) when donor was younger or equal to the indicated age (**B**) when donor was older or equal to the indicated age. Trough in (**A**) and peak in (**B**) are at 40 and 41 y (dotted grid line), validating donor age of 40 y for optimum stratification. A part of (**A**) was magnified for better visualization.

First, as shown in Figure 3A, we analyzed how significantly graft failure risk was different between female-to-male LT and other donor-recipient sex groups when donors were younger or equal to a certain age. The degree of statistical significance increased until the donor age of 40 y and 41 y with the trough *p*-value of 0.003. Second, as shown in Figure 3B, we analyzed the significance of sex disparity when donors were older or equal to a certain age. The degree of sex disparity was already insignificant at 16 y (the whole cohort) with a *p*-value of 0.216. The degree of statistical significance gradually decreased until the age of 40 y and 41 y with peak *p*-values of 0.999 and 0.989, respectively. These results determined that donor age 40 y is the best cutoff age to define the risk subgroup of recipients with a significant impact of female-to-male donation.

## 4. Discussion

In the current study analyzing the UNOS OPTN database, we found that the risk of graft failure after deceased donor LT is greater in female-to-male donation only when donor age is ≤40 y, as previously reported in living donor LT [10,18], and graft failure risk is comparable irrespective of donor-recipient sex combinations when donor age is >40 y. A previous study demonstrated that the tolerance to hepatic ischemia-reperfusion injury is greater in females than in males only when the age is ≤40 y and hepatic estrogen receptor expression is also greater in females only when the age is ≤40 y [6]. Our research team also demonstrated that the risk of graft failure risk is greater in female-to-male LT only when living donor age is ≤40 y and the female graft is positive at estrogen receptor expression [18]. Continuing from the series studies of living donors and their recipients, the current study of deceased-donor-LT recipients demonstrated that greater graft failure risk in female-to-male LT is found only when donor is ≤40 y. Despite young donor age of ≤40 y, which indicates good graft parenchymal quality, graft failure risk of female-to-male LT reached the level of recipients with >40 y donors, suggesting the presence of another contributor for graft failure that outweighs donor age in terms of graft quality. Comparable graft failure risk irrespective of donor-recipient sex combinations with >40 y donors further suggests that anatomical size mismatch between female donor and male recipient regarding vessel, bile duct, and graft size, is not the reason for the inferiority of female-to-male LT, being in agreement with our recent research [10].

Although the exact mechanisms underlying the poorer outcome of female-to-male LT is unknown, hepatic estrogen signaling is considered to play an important role [17,25]. Hepatic estrogen signaling is positively correlated with hepatic protection capacity [6,9,22,25,26]. A previous study demonstrated that all female mice survived after partial hepatic inflow occlusion for 45 min, whereas all male mice died with greater hepatocyte injury [9]. In the study, ovariectomy or estrogen antagonist administration to female mice increased hepatocyte injury, reduced liver regeneration, and increased mortality, whereas estradiol administration to male mice improved recovery [9]. In rats, translocation of estrogen receptors from the cytosol to nucleus and activation of cell signaling occurred after liver injury to stimulate the healing process [27,28]. Our recent study of healthy living liver donors has also demonstrated that the tolerance to hepatic ischemia-reperfusion injury is greater in females, and the protective capacity is correlated with hepatic estrogen receptor expression level [6]. Thus, it has been hypothesized that acute post-transplant decrease in hepatic estrogen receptor expression, which is specifically observed in female-to-male LT, is a reason of graft failure [13,14,16]. The abrogation of the inferiority of female-to-male LT when the donor was >40 y in our previous study of living-donor-LT recipient [18] and the current study of deceased-donor-LT recipients support the involvement of estrogen receptor in the risk of female-to-male donation because hepatic estrogen receptor activity changes by the age of 40 y [10,29]. Despite the significantly high graft failure risk of female-to-male LT, previous studies have not given sufficient efforts to evaluate the exact underlying mechanisms or simply deduced that the inferiority is due to the graft size mismatch. Since the recent evidence has consistently suggested that the inferiority of female-to-male LT does not simply result from graft size mismatch, future research should elucidate another mechanisms underlying its poorer outcome. 

To generate a homogeneous study population, we excluded recipients diagnosed as HCC and those with macrosteatotic grafts because donor macrosteatosis and recipient HCC were reported to modify the effects of female-to-male donation. In particular, a different impact of donor-recipient sex combination on post-transplant outcome was reported between HCC patients and non-HCC patients [7,10,18]. A previous study demonstrated that HCC recurrence risk was greater in recipients with male donors than those with female donors regarding intrahepatic recurrence and extrahepatic recurrence, and this finding was externally validated by analyzing the UNOS data [7]. In particular, the significance of male donor was robust within male recipient, indicating greater HCC recurrence after male-to-male LT. The poorer outcome of male donor LT was abrogated when donor age was > 40 y, being in line with the current study. The poorer outcome of recipients with male donor and male-to-male LT was validated by a very recent study analyzing the European Liver Transplant Registry (ELTR) database demonstrating the greater HCC-related death risk after male-to-male LT [19].

This study has several limitations. First, due to the retrospective nature, we were unable to exclude the possibility of bias from unmeasured variables. Particularly, perioperative factors which were significant in our previous studies (e.g., neutrophil-to-lymphocyte ratio and red blood cell transfusion) could not be analyzed due to the lack of data in the UNOS OPTN database [10,18,30]. Immunosuppression protocol could not be analyzed due to the same reason. Also, large subject loss (more than 70,000 recipients) occurred due to missing graft macrosteatosis degree. However, the inferiority of female-to-male LT was also shown even before excluding the patients with missing biopsy data (Supplementary Figure S1). In addition, the underlying causes of recipient death could not be analyzed because it was recorded for only 2989 patients in the whole cohort and 900 patients among patients with ≤40 y donors. If the underlying causes of death after female-to-male LT are analyzed as performed in living donor LT [10], it would help elucidate the underlying mechanisms of the inferiority of female-to-male LT. Second, the cutoff value of 40 y may not be absolute as our previous research in living donor LT determined that donor age between 36 y and 41 y is the time window to define the risk subgroup in female-to-male LT [10,29]. Nonetheless, the current study with a large cohort of deceased donor LT determined that 40 y is the best cutoff donor age. Third, we could not examine the biopsied graft tissue to analyze hepatic estrogen receptor expression and its association with the impact of female-to-male LT that was possible in our previous research [6,18]. Fourth, more specific subgroups modifying the impact of female-to-male LT should be studied in future research, potentially in the context of pre-/post-transplant hepatic estrogen receptor activity, which may differ by various donor/recipient factors along with donor age. Graft allocation is ethically sensitive and complex with organ shortage and inevitable competition. More scientific data compensating above-mentioned limitations will help imply the inferiority of female-to-male LT in graft allocation system.

In the current study analyzing recipients who underwent deceased donor LT and registered in the UNOS OPTN database, we once again validated that the graft failure risk is greater in female-to-male LT than other donor-recipient sex combinations. Of importance, we for the first time found that the inferiority of female-to-male LT is found only when donor age ias ≤40 y in deceased donor LT. That is, graft failure risk was comparable irrespective of donor-recipient sex combinations when deceased donor age was >40 y. Also, we determined that 40 y is the best cutoff donor age to define the risk subgroup of recipients in female-to-male LT. These findings are consistent with the results obtained from living donor LT in previous research.

## Figures and Tables

**Table 1:** Clinical data of recipients according to donor–recipient sex in subgroup with donor ≤ 40 y.

	Female to Male (*n* = 920)	Female to Female (*n* = 780)	Male to Female (*n* = 828)	Male to Male (*n* = 1602)
Donor age (y)	32 (25–37)	32 (25–36)	29 (22–35)	30 (24–35)
Donor body mass index (kg/m^2^)	28.1 (23.7–33.9)	26.4 (22.7–31.6)	25.9 (23.0–29.5)	27.6 (24.2–31.7)
Donor cause of death				
Anoxia	516 (58.0)	431 (57.2)	398 (49.8)	771 (49.6)
Cerebrovascular/stroke	190 (21.4)	165 (21.9)	76 (9.5)	174 (11.2)
Others *	214 (23.3)	184 (23.6)	354 (42.8)	657 (41.0)
Cold ischemia time (h)	6.2 (5.0–8.0)	6.2 (5.0–8.0)	6.2 (5.0–7.6)	6.3 (5.0–8.0)
Transplant era				
2000–2007	119 (12.9)	102 (13.1)	87 (10.4)	212 (12.2)
2008–2015	277 (30.1)	206 (26.4)	244 (29.5)	462 (28.8)
2016–2023	524 (57.0)	472 (60.5)	498 (60.1)	945 (59.0)
Age (y)	54 (47–60)	55 (44–62)	55 (45–61)	54 (46–60)
Body mass index (kg/m^2^)	27.5 (23.8–31.3)	27.4 (23.4–32.3)	27.9 (23.8–33.0)	28.1 (24.4–32.1)
Diabetes	236 (25.7)	202 (25.9)	192 (23.2)	392 (24.5)
Primary liver disease				
Viral	211 (22.9)	134 (17.2)	122 (14.7)	343 (21.4)
Alcoholic	356 (38.7)	171 (21.9)	221 (26.7)	635 (39.6)
MASH	114 (12.4)	140 (17.9)	153 (18.5)	213 (13.3)
Others ^†^	239 (26.0)	335 (42.9)	332 (40.1)	411 (25.7)
Hepatic encephalopathy III–IV	132 (14.4)	103 (13.2)	125 (15.1)	217 (13.6)
Refractory ascites	359 (39.1)	271 (34.7)	305 (36.8)	650 (40.6)
Previous upper abdominal surgery	374 (40.7)	458 (58.7)	475 (57.4)	605 (37.8)
Portal vein thrombosis	100 (10.9)	87 (11.2)	94 (11.4)	162 (10.1)
MELD score	26 (19–33)	25 (19–33)	27 (20–34)	25 (19–33)
Pretransplant critical care				
Dialysis	180 (19.6)	154 (19.7)	194 (23.4)	289 (18.0)
Life-supporting device	78 (8.5)	68 (8.7)	102 (12.3)	151 (9.4)
Mechanical ventilation	40 (4.3)	35 (4.5)	62 (7.5)	74 (4.6)
Pretransplant biochemical status				
Albumin (g/dL)	3.0 (2.6–3.4)	3.0 (2.6–3.5)	3.1 (2.6–3.6)	3.0 (2.6–3.5)
Total bilirubin (mg/dL)	5.5 (2.6–15.2)	5.6 (2.6–14.3)	7.1 (2.7–17.8)	5.3 (2.3–13.7)
Creatinine (mg/dL)	1.30 (0.90–2.17)	1.10 (0.80–1.91)	1.13 (0.80–2.00)	1.30 (0.89–2.10)
Prothrombin time (INR)	1.80 (1.50–2.40)	1.80 (1.40–2.40)	1.90 (1.40–2.60)	1.80 (1.40–2.40)

Data are presented as median (interquartile range) or frequency (%). INR, international normalized ratio. MASH, metabolic dysfunction-associated steatohepatitis; MELD, model for end-stage liver disease. * Cause of death of donors is categorized into five groups in the UNOS OPTN data: anoxia, cerebrovascular/stroke, head trauma, central nervous system tumor, and others. Because the frequency of the last three categories is too small to analyze, they were combined into one value “Others”. ^†^ See Supplementary Table S1.

**Table 2:** Clinical data of recipients according to donor–recipient sex in subgroup with donor > 40 y.

	Female to Male (*n* = 1984)	Female to Female (*n* = 1676)	Male to Female (*n* = 1271)	Male to Male (*n* = 2691)
Donor age (y)	54 (48–63)	56 (48–64)	55 (49–63)	55 (48–62)
Donor body mass index (kg/m^2^)	28.1 (23.7–33.9)	26.4 (22.7–31.6)	25.9 (23.0–29.5)	27.6 (24.2–31.7)
Donor cause of death				
Anoxia	651 (33.3)	491 (29.9)	392 (31.4)	859 (32.5)
Cerebrovascular/stroke	1118 (57.2)	953 (58.1)	599 (47.9)	1213 (45.9)
Others *	215 (10.8)	232 (13.8)	280 (22.0)	619 (23.0)
Cold ischemia time (h)	6.3 (5.0–8.0)	6.1 (4.9–7.8)	6.0 (4.8–7.7)	6.2 (5.0–8.0)
Transplant era				
2000–2007	325 (15.4)	237 (14.1)	170 (13.4)	408 (15.2)
2008–2015	757 (38.2)	609 (36.3)	396 (31.2)	864 (32.1)
2016–2023	902 (45.5)	830 (49.5)	705 (55.5)	1419 (52.7)
Age (y)	55 (48–61)	56 (48–63)	56 (48–62)	55 (48–61)
Body mass index (kg/m^2^)	27.7 (24.2–32.1)	27.5 (23.7–32.5)	28.1 (24.2–32.4)	28.3 (24.9–32.3)
Diabetes	505 (25.5)	422 (25.2)	344 (27.1)	702 (26.1)
Primary liver disease				
Viral	412 (20.8)	246 (14.7)	199 (15.7)	544 (20.2)
Alcoholic	722 (36.4)	372 (22.2)	301 (23.7)	1062 (39.5)
MASH	287 (14.5)	337 (20.1)	269 (21.2)	396 (14.7)
Others ^†^	563 (28.4)	721 (43.0)	502 (39.5)	689 (25.6)
Hepatic encephalopathy III–IV	277 (14.0)	256 (15.3)	201 (15.8)	378 (14.1)
Refractory ascites	772 (38.9)	576 (34.4)	445 (35.0)	1064 (39.6)
Previous upper abdominal surgery	784 (39.5)	975 (58.2)	770 (60.6)	1024 (38.1)
Portal vein thrombosis	138 (12.0)	191 (11.4)	147 (11.6)	323 (12.0)
MELD score	24 (18–31)	24 (18–31)	24 (18–31)	23 (18–30)
Pretransplant critical care				
Dialysis	254 (12.8)	253 (15.1)	206 (16.2)	327 (12.2)
Life-supporting device	135 (6.8)	160 (9.6)	106 (8.3)	193 (7.2)
Mechanical ventilation	86 (4.3)	103 (6.1)	64 (5.0)	92 (3.4)
Pretransplant biochemical status				
Albumin (g/dL)	3.0 (2.5–3.5)	3.0 (2.5–3.5)	3.0 (2.6–3.5)	3.0 (2.5–3.5)
Total bilirubin (mg/dL)	5.4 (2.6–12.9)	5.4 (2.5–13.4)	5.3 (2.5–12.5)	4.5 (2.3–10.5)
Creatinine (mg/dL)	1.24 (0.90–1.90)	1.07 (0.72–1.70)	1.10 (0.79–1.75)	1.20 (0.90–1.83)
Prothrombin time (INR)	1.80 (1.44–2.30)	1.80 (1.40–2.40)	1.80 (1.40–2.32)	1.78 (1.40–2.30)

Data are presented as median (interquartile range) or frequency (%). INR, international normalized ratio; MASH, metabolic dysfunction-associated steatohepatitis; MELD, model for end-stage liver disease. * Cause of death of donors is categorized into five groups in the UNOS OPTN data: anoxia, cerebrovascular/stroke, head trauma, central nervous system tumor, and others. Because the frequency of the last three categories is too small to analyze, they were combined into one value “Others”. ^†^ See Supplementary Table S1.

**Table 3:** Multivariable analysis for graft failure in subgroup of recipients with donor ≤ 40 y.

	Hazard Ratio	*p*-Value
Female-to-male transplantation		
vs. Female-to-female	1.48 (1.17–1.81)	<0.001
vs. Male-to-female	1.51 (1.21–1.89)	<0.001
vs. Male-to-male	1.27 (1.06–1.52)	0.009
Donor age (y)	1.00 (0.99–1.01)	0.367
Donor body mass index (kg/m^2^)	1.00 (0.99–1.01)	0.410
Cause of donor death (vs. anoxia)		
Cerebrovascular/stroke	1.33 (1.09–1.61)	0.004
Others	1.02 (0.99–1.04)	0.184
Cold ischemia time (h)	1.02 (0.99–1.04)	0.156
Transplant era (vs. 2000–2007)		0.001
2008–2015	0.73 (0.60–0.88)	<0.001
2016–2023	0.70 (0.56–0.86)	0.001
Age (y)	1.02 (1.01–1.02)	<0.001
Body mass index (kg/m^2^)	0.98 (0.97–0.99)	0.002
Diabetes	1.39 (1.19–1.62)	<0.001
Primary liver disease (vs. viral)		
Alcoholic	1.03 (0.84–1.25)	0.803
MASH	1.10 (0.86–1.42)	0.439
Others	1.22 (1.01–1.47)	0.037
Hepatic encephalopathy III–IV	1.16 (0.94–1.42)	0.163
Refractory ascites	1.13 (0.97–1.32)	0.105
Previous upper abdominal surgery	1.20 (1.04–1.38)	0.013
Portal vein thrombosis	1.15 (0.92–1.43)	0.229
MELD score	0.99 (0.98–1.01)	0.352
Dialysis	1.23 (0.98–1.55)	0.123
Life-supporting device	1.20 (0.84–1.70)	0.311
Mechanical ventilation	1.19 (0.78–1.83)	0.423
Albumin (g/dL)	0.99 (0.90–1.09)	0.815
Total bilirubin (mg/dL)	1.01 (1.00–1.02)	0.105
Creatinine (mg/dL)	1.02 (0.97–1.07)	0.551
Prothrombin time (INR)	1.02 (0.96–1.08)	0.484

INR, international normalized ratio; MASH, model for end-stage liver disease; MELD, model for end-stage liver disease.

**Table 4:** Multivariable analysis for graft failure in subgroup of recipients with donor > 40 y.

	Hazard Ratio	*p*-Value
Female-to-male transplantation		
vs. Female-to-female	1.00 (0.88–1.14)	0.981
vs. Male-to-female	0.98 (0.85–1.13)	0.758
vs. Male-to-male	0.97 (0.86–1.08)	0.551
Donor age (y)	1.01 (1.01–1.01)	<0.001
Donor body mass index (kg/m^2^)	1.00 (0.99–1.00)	0.507
Cause of donor death (vs. anoxia)		
Cerebrovascular/stroke	1.09 (0.97–1.21)	0.141
Others	0.92 (0.80–1.06)	0.238
Cold ischemia time (h)	1.02 (1.01–1.04)	0.002
Transplant era (vs. 2000–2007)		<0.001
2008–2015	0.76 (0.67–0.84)	<0.001
2016–2023	0.60 (0.52–0.69)	<0.001
Age (y)	1.00 (1.00–1.01)	0.522
Body mass index (kg/m^2^)	1.00 (0.99–1.01)	0.693
Diabetes	1.28 (1.16–1.42)	<0.001
Primary liver disease (vs. viral)		
Alcoholic	1.00 (0.89–1.14)	0.960
MASH	0.86 (0.74–1.01)	0.071
Others	0.89 (0.79–1.00)	0.056
Hepatic encephalopathy III-IV	1.07 (0.94–1.22)	0.321
Refractory ascites	1.02 (0.93–1.12)	0.713
Previous upper abdominal surgery	1.24 (1.13–1.36)	<0.001
Portal vein thrombosis	1.25 (1.10–1.43)	<0.001
MELD score	1.01 (1.00–1.02)	0.237
Dialysis	1.16 (0.99–1.37)	0.067
Life-supporting device	1.17 (0.89–1.52)	0.261
Mechanical ventilation	1.59 (1.17–2.15)	0.003
Albumin (g/dL)	0.90 (0.84–0.95)	<0.001
Total bilirubin (mg/dL)	1.00 (0.99–1.01)	0.856
Creatinine (mg/dL)	1.06 (1.02–1.10)	0.005
Prothrombin time (INR)	0.97 (0.93–1.02)	0.277

INR, international normalized ratio; MASH, model for end-stage liver disease; MELD, model for end-stage liver disease.

## Data Availability

The data have been supplied by the UNOS as the contractor for the OPTN. The database used and/or analyzed during the current study is available from the corresponding author on reasonable request.

## Supplementary Materials

The following supporting information can be downloaded at: https://www.mdpi.com/article/10.3390/jpm15080357/s1, Figure S1: Analysis including subjects with missing macrosteatosis degree; Table S1: Etiology categorization.

## Abbreviations

The following abbreviations are used in this manuscript:

ELTR	European liver transplant registry
HCC	hepatocellular carcinoma
HR	hazard ratio
LT	liver transplantation
MASH	metabolic dysfunction-associated steatohepatitis
OPTN	Organ Procurement and Transplantation Network
UNOS	United Network for Organ Sharing

## References

[1] Shi Y., Ma J., Li S., Liu C., Liu Y., Chen J., Liu N., Liu S., Huang H. (2024). Sex difference in human diseases: Mechanistic insights and clinical implications. Signal Transduct. Target. Ther..

[2] Feng Z., Liao M., Zhang L. (2024). Sex differences in disease: Sex chromosome and immunity. J. Transl. Med..

[3] Massey S.C., Whitmire P., Doyle T.E., Ippolito J.E., Mrugala M.M., Hu L.S., Canoll P., Anderson A.R.A., Wilson M.A., Fitzpatrick S.M. (2021). Sex differences in health and disease: A review of biological sex differences relevant to cancer with a spotlight on glioma. Cancer Lett..

[4] Cattaneo A., Bellenghi M., Ferroni E., Mangia C., Marconi M., Rizza P., Borghini A., Martini L., Luciani M.N., Ortona E. (2024). Recommendations for the Application of Sex and Gender Medicine in Preclinical, Epidemiological and Clinical Research. J. Pers. Med..

[5] El-Serag H.B., Rudolph K.L. (2007). Hepatocellular carcinoma: Epidemiology and molecular carcinogenesis. Gastroenterology.

[6] Han S., Cho J., Wi W., Won Lee K., Hwa Cha H., Lee S., Hyun Ahn J., Kim S., Sung Choi G., Man Kim J. (2022). Sex difference in the tolerance of hepatic ischemia-reperfusion injury and hepatic estrogen receptor expression according to age and macrosteatosis in healthy living liver donors. Transplantation.

[7] Han S., Yang J.D., Sinn D.H., Kim J.M., Choi G.S., Jung G., Ahn J.H., Kim S., Ko J.S., Gwak M.S. (2018). Risk of post-transplant hepatocellular carcinoma recurrence is higher in recipients of livers from male than female living donors. Ann. Surg..

[8] Kasarinaite A., Sinton M., Saunders P.T.K., Hay D.C. (2023). The Influence of Sex Hormones in Liver Function and Disease. Cells.

[9] Harada H., Pavlick K.P., Hines I.N., Hoffman J.M., Bharwani S., Gray L., Wolf R.E., Grisham M.B. (2001). Effects of gender on reduced-size liver ischemia and reperfusion injury. J. Appl. Physiol..

[10] Lee K.W., Han S., Lee S., Cha H.H., Ahn S., Ahn H.S., Ko J.S., Gwak M.S., Kim G.S., Joh J.W. (2018). Higher risk of posttransplant liver graft failure in male recipients of female donor grafts might not be due to anastomotic size disparity. Transplantation.

[11] Kahn D., Gavaler J.S., Makowka L., van Thiel D.H. (1993). Gender of donor influences outcome after orthotopic liver transplantation in adults. Dig. Dis. Sci..

[12] Brooks B.K., Levy M.F., Jennings L.W., Abbasoglu O., Vodapally M., Goldstein R.M., Husberg B.S., Gonwa T.A., Klintmalm G.B. (1996). Influence of donor and recipient gender on the outcome of liver transplantation. Transplantation.

[13] Francavilla R., Hadzic N., Heaton N.D., Rela M., Baker A.J., Dhawan A., Mieli-Vergani G. (1998). Gender matching and outcome after pediatric liver transplantation. Transplantation.

[14] Croome K.P., Segal D., Hernandez-Alejandro R., Adams P.C., Thomson A., Chandok N. (2014). Female donor to male recipient gender discordance results in inferior graft survival: A prospective study of 1,042 liver transplants. J. Hepatobiliary Pancreat. Sci..

[15] Gao F., Dong L., Chen J., Xu S., Wang Z., Xu H., Qiu X., Wu Y., Shao C., Wei X. (2025). Impact of donor-recipient sex-matching patterns on liver transplantation outcomes: A cohort study based on United Network of Organ Sharing data. Hepatobiliary Surg. Nutr..

[16] Yoshizumi T., Shirabe K., Taketomi A., Uchiyama H., Harada N., Ijichi H., Yoshimatsu M., Ikegami T., Soejima Y., Maehara Y. (2012). Risk factors that increase mortality after living donor liver transplantation. Transplantation.

[17] Kahn D., Zeng Q.H., Makowka L., Murase N., Nakajima Y., Eagon P.K., Francavilla A., Starzl T.E., Van Thiel D.H. (1989). Orthotopic liver transplantation and the cytosolic estrogen-androgen receptor status of the liver: The influence of the sex of the donor. Hepatology.

[18] Han S., Kwon J.H., Lee K.W., Lee S., Choi G.S., Kim J.M., Ko J.S., Gwak M.S., Kim G.S., Ha S.Y. (2023). Abrogation of greater graft failure risk of female-to-male liver transplantation with donors older than 40 years or graft macrosteatosis greater than 5%. Sci. Rep..

[19] Magyar C.T.J., Arteaga N.F., Germani G., Karam V.H., Adam R., Romagnoli R., De Simone P., Robin F., Cherqui D., Bosca A. (2024). Recipient-donor sex constellation in liver transplantation for hepatocellular carcinoma-an ELTR study. Liver Int. Off. J. Int. Assoc. Study Liver.

[20] Kleiner D.E., Brunt E.M., Van Natta M., Behling C., Contos M.J., Cummings O.W., Ferrell L.D., Liu Y.C., Torbenson M.S., Unalp-Arida A. (2005). Design and validation of a histological scoring system for nonalcoholic fatty liver disease. Hepatology.

[21] Tannapfel A., Denk H., Dienes H.P., Langner C., Schirmacher P., Trauner M., Flott-Rahmel B. (2011). Histopathological diagnosis of non-alcoholic and alcoholic fatty liver disease. Virchows Arch. Int. J. Pathol..

[22] Erkan G., Yilmaz G., Konca Degertekin C., Akyol G., Ozenirler S. (2013). Presence and extent of estrogen receptor-alpha expression in patients with simple steatosis and NASH. Pathol. Res. Pract..

[23] Han S., Kim G., Lee S.K., Kwon C.H., Gwak M., Lee S., Ha S., Park C.K., Ko J.S., Joh J. (2014). Comparison of the tolerance of hepatic ischemia/reperfusion injury in living donors: Macrosteatosis versus microsteatosis. Liver Transpl..

[24] Galle P.R., Forner A., Llovet J.M., Mazzaferro V., Piscaglia F., Raoul J.L., European Association for the Study of the Liver (2024). EASL-EASD-EASO Clinical Practice Guidelines on the management of metabolic dysfunction-associated steatotic liver disease (MASLD). J. Hepatol..

[25] Eagon P.K., Porter L.E., Francavilla A., DiLeo A., Van Thiel D.H. (1985). Estrogen and androgen receptors in liver: Their role in liver disease and regeneration. Semin. Liver Dis..

[26] Chen K.L., Madak-Erdogan Z. (2018). Estrogens and female liver health. Steroids.

[27] Francavilla A., di Leo A., Eagon P.K., Wu S.Q., Ove P., van Thiel D.H., Starzl T.E. (1984). Regenerating rat liver: Correlations between estrogen receptor localization and deoxyribonucleic acid synthesis. Gastroenterology.

[28] Fisher B., Gunduz N., Saffer E.A., Zheng S. (1984). Relation of estrogen and its receptor to rat liver growth and regeneration. Cancer Res..

[29] Faddy M.J., Gosden R.G., Gougeon A., Richardson S.J., Nelson J.F. (1992). Accelerated disappearance of ovarian follicles in mid-life: Implications for forecasting menopause. Hum. Reprod..

[30] Han S., Kwon J.H., Jung S.H., Seo J.Y., Jo Y.J., Jang J.S., Yeon S.M., Jung S.H., Ko J.S., Gwak M.S. (2018). Perioperative fresh red blood cell transfusion may negatively affect recipient survival after liver transplantation. Ann. Surg..

