# Supplementary material for: Impact of Donor Age on Graft Failure After Deceased Donor Liver Transplantation by Donor-Recipient Sex Combinations: An Analysis of the UNOS OPTN Database
Source: J Pers Med. 2025 Aug 5;15(8):357. doi: 10.3390/jpm15080357 (PMC12387996; doi:10.3390/jpm15080357)
Supplement: Supplementary file 1 [file jpm-15-00357-s001.zip › jpm-3679612-supplementary.pdf]

Table S1: Etiology categorization

TABLE S1	Classified as	Final grouping	Code
Other specify	Miscellaneous	Others	999
AHN: DRUG OTHER SPECIFY	Drug-induced	Others	4100
AHN: TYPE A	Viral	Viral	4101
AHN: TYPE B- HBSAG+	Viral	Viral	4102
AHN: TYPE C	Viral	Viral	4104
AHN: TYPE B AND C	Viral	Viral	4106
AHN: TYPE B AND D	Viral	Viral	4107
AHN: ETIOLOGY UNKNOWN	Cryptogenic	Others	4108
AHN: OTHER, SPECIFY (E.G., ACUTE VIRAL INFECTION, AUTOIMMUNE HEPATITIS - FULMINANT)	Miscellaneous	Others	4110
CIRRHOSIS: DRUG/INDUST EXPOSURE OTHER SPECIFY	Drug-induced	Others	4200
CIRRHOSIS: TYPE A	Viral	Viral	4201
CIRRHOSIS: TYPE B- HBSAG+	Viral	Viral	4202
CIRRHOSIS: TYPE C	Viral	Viral	4204
CIRRHOSIS: TYPE D	Viral	Viral	4205
CIRRHOSIS: TYPE B AND C	Viral	Viral	4206
CIRRHOSIS: TYPE B AND D	Viral	Viral	4207
CIRRHOSIS: CRYPTOGENIC- IDIOPATHIC	Cryptogenic	Others	4208
CIRRHOSIS: CHRONIC ACTIVE HEPATITIS: ETIOLOGY UNKNOWN	Cryptogenic	Others	4209
CIRRHOSIS: OTHER, SPECIFY (E.G., HISTIOCYTOSIS, SARCOIDOSIS, GRANULOMATOUS)	Miscellaneous	Others	4210
CIRRHOSIS: AUTOIMMUNE	Autoimmune	Others	4212
CIRRHOSIS: CRYPTOGENIC (IDIOPATHIC)	Cryptogenic	Others	4213
CIRRHOSIS: FATTY LIVER (NASH)	MASH	MASH	4214
ALCOHOLIC CIRRHOSIS	Alcoholic	Alcoholic	4215
ALCOHOLIC CIRRHOSIS WITH HEPATITIS C	Alcoholic	Alcoholic	4216
ACUTE ALCOHOLIC HEPATITIS	Alcoholic	Alcoholic	4217

ACUTE ALCOHOL-ASSOCIATED HEPATITIS WITH OR WITHOUT CIRRHOSIS	Alcoholic	Alcoholic	4218
ALCOHOL-ASSOCIATED CIRRHOSIS WITHOUT ACUTE ALCOHOL-ASSOCIATED HEPATITIS	Alcoholic	Alcoholic	4219
PRIMARY BILIARY CIRRHOSIS (PBC)	Biliary	Others	4220
SEC BILIARY CIRRHOSIS: CAROLI'S DISEASE	Biliary	Others	4230
SEC BILIARY CIRRHOSIS: CHOLEDOCHOL CYST	Biliary	Others	4231
SEC BILIARY CIRRHOSIS: OTHER SPECIFY	Biliary	Others	4235
PSC: CROHN'S DISEASE	Biliary	Others	4240
PSC: ULCERATIVE COLITIS	Biliary	Others	4241
PSC: NO BOWEL DISEASE	Biliary	Others	4242
PSC: OTHER SPECIFY	Biliary	Others	4245
FAMILIAL CHOLESTASIS: OTHER SPECIFY	Biliary	Others	4250
CHOLES LIVER DISEASE: OTHER SPECIFY	Biliary	Others	4260
NEONATAL HEPATITIS OTHER SPECIFY	Others	Others	4265
BILIARY ATRESIA: EXTRAHEPATIC	Biliary	Others	4270
BILIARY HYPOPLASIA: NONSYNDROMIC PAUCITY OF INTRAHEPATIC BILE DUCT	Biliary	Others	4271
BILIARY HYPOPLASIA: ALAGILLE’S SYNDROME (PAUCITY OF INTRAHEPATIC BILE DUCT)	Biliary	Others	4272
BILIARY ATRESIA OR HYPOPLASIA: OTHER, SPECIFY	Biliary	Others	4275
CONGENITAL HEPATIC FIBROSIS	Miscellaneous	Others	4280
CYSTIC FIBROSIS	Miscellaneous	Others	4285
BUDD-CHIARI SYNDROME	Miscellaneous	Others	4290
METDIS: ALPHA-1-ANTITRYPSIN DEFIC A-1-A	Metabolic	Others	4300

METDIS: WILSON'S DISEASE, OTHER COPPER METABOLISM DISORDER	Metabolic	Others	4301
METDIS: HEMOCHROMATOSIS - HEMOSIDEROSIS	Metabolic	Others	4302
METDIS: GLYC STOR DIS TYPE I (GSD-I)	Metabolic	Others	4303
METDIS: HYPERLIPIDEMIA-II, HOMOZYGOUS HYPERCHOLESTEROLEMIA	Metabolic	Others	4305
METDIS: PRIMARY OXALOSIS/OXALURIA, HYPEROXALURIA	Metabolic	Others	4307
METDIS: MAPLE SYRUP URINE DISEASE	Metabolic	Others	4308
METDIS: OTHER SPECIFY	Metabolic	Others	4315
PLM: FIBROLAMELLAR (FL-HC)	Tumor other than HCC	Others	4402
PLM: CHOLANGIOCARCINOMA (CH-CA)	Tumor other than HCC	Others	4403
PLM: HEMANGIOENDOTHELIOMA, HEMANGIOSARCOMA, ANGIOSARCOMA	Tumor other than HCC	Others	4405
PLM: OTHER SPECIFY (I.E., KLATZKIN TUMOR, LEIOMYSARCOMA)	Tumor other than HCC	Others	4410
BILE DUCT CANCER: (CHOLANGIOMA, BILIARY TRACT CARCINOMA)	Tumor other than HCC	Others	4420
SECONDARY HEPATIC MALIGNANCY OTHER SPECIFY	Tumor other than HCC	Others	4430
BENIGN TUMOR: HEPATIC ADENOMA	Tumor other than HCC	Others	4450
BENIGN TUMOR: POLYCYSTIC LIVER DISEASE	Tumor other than HCC	Others	4451
BENIGN TUMOR: OTHER SPECIFY	Tumor other than HCC	Others	4455
TPN/HYPERALIMENTATION IND LIVER DISEASE	Miscellaneous	Others	4500
TRAUMA OTHER SPECIFY	Miscellaneous	Others	4520

Figure S1: Analysis including subjects with missing macrosteatosis degree

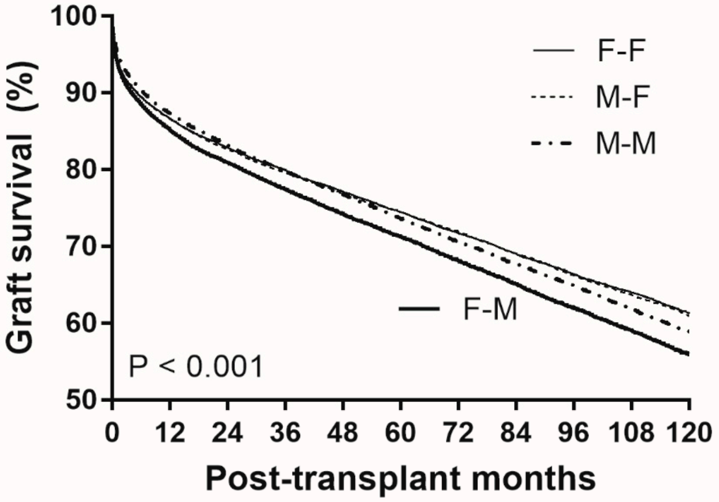

Subjects at risk											
F-M	24403	19904	17265	14926	13107	11550	10077	8740	7537	6543	5590
F-F	19347	15970	13806	11889	10414	9119	7942	6929	6072	5314	4583
M-F	21462	17701	14996	13092	11404	10035	8732	7522	6514	5638	4833
M-M	43784	36499	31536	27343	23921	20892	18185	15808	13838	11982	10398

Hazard Ratio

F-M vs. F-F 1.16 (1.13-1.21)

F-M vs. M-F 1.16 (1.12-1.19)

F-M vs. M-M 1.11 (1.08-1.14)
